# Transcriptome Profile in Unilateral Adrenalectomy-Induced Compensatory Adrenal Growth in the Rat

**DOI:** 10.3390/ijms19041111

**Published:** 2018-04-07

**Authors:** Karol Jopek, Marianna Tyczewska, Piotr Celichowski, Ludwik K. Malendowicz, Marcin Rucinski

**Affiliations:** 1 Department of Histology and Embryology, Poznan University of Medical Sciences, Swiecickiego 6 Street, 60-781 Poznan, Poland; kjopek@ump.edu.pl (K.J.); maritycz@ump.edu.pl (M.T.); pcelichowski@ump.edu.pl (P.C.); lkm@ump.edu.pl (L.K.M.); 2 Department of Anatomy and Histology, University of Zielona Gora, Zyty 28 Street, 65-046 Zielona Gora, Poland

**Keywords:** rat, adrenal gland, gene expression profile, compensatory adrenal growth, unilateral adrenalectomy, gene ontology

## Abstract

Compensatory adrenal growth evoked by unilateral adrenalectomy (hemiadrenalectomy) constitutes one of the most frequently studied in vivo models of adrenocortical enlargement. This type of growth has been quite well characterized for its morphological, biochemical, and morphometric parameters. However, the molecular basis of compensatory adrenal growth is poorly understood. Therefore, the aim of this study was to investigate the rat adrenal transcriptome profile during the time of two previously described adrenocortical proliferation waves at 24 and 72 h after unilateral adrenalectomy. Surgical removal of the left adrenal or a sham operation was accomplished via the classic dorsal approach. As expected, the weight of the remaining right adrenal glands collected at 24 and 72 h after hemiadrenalectomy increased significantly. The transcriptome profile was identified by means of Affymetrix^®^ Rat Gene 2.1 ST Array. The general profiles of differentially expressed genes were visualized as volcano plots and heatmaps. Detailed analyzes consisted of identifying significantly enriched gene ontological groups relevant to adrenal physiology, by means of DAVID and GOplot bioinformatics tools. The results of our studies showed that compensatory adrenal growth induced by unilateral adrenalectomy exerts a limited influence on the global transcriptome profile of the rat adrenal gland; nevertheless, it leads to significant changes in the expression of key genes regulating the circadian rhythm. Our results confirm also that regulation of compensatory adrenal growth is under complex and multifactorial control with a pivotal role of neural regulatory mechanisms and a supportive role of other components.

## 1. Introduction

Adrenal growth, particularly its cortical compartment, has been the subject of numerous studies for years. The specific reactivity of this gland to various endogenous and exogenous factors ensures maintenance of the organism homeostasis, for which the adrenocorticosteroid hormones are responsible. Maintaining proper homeostasis, especially in the case of longer periods, is usually associated with changes in adrenal mass, mainly its enlargement. These changes are caused by the proliferation (hyperplasia) and/or hypertrophy of adrenocortical cells. As known, the main factor regulating adrenal cortex growth in vivo is ACTH. However, in addition to ACTH, a number of other humoral factors, as well as the nervous system, are involved in the regulation of gland growth. It should be underlined that adrenal cortex growth disturbances resulting in hypertrophy or hyperplasia of the gland may lead to the development of adrenal tumors.

In 1984, Dallman, in her excellent monograph, described various types of adrenal cortex growth in vivo and described the main factors regulating them [1]. Among others, the author distinguishes the following types of adrenal growth: (I) growth correlated with age (in relation to body weight), (II) growth induced by elevated concentrations of ACTH, (III) enucleation-induced adrenal regeneration, and (IV) compensatory adrenal growth induced by unilateral adrenalectomy. In numerous species (mouse, rat, hamster, guinea pig), the above-mentioned growth types of the adrenal cortex are well characterized by morphological, biochemical, and morphometric methods. However, the genetic basis of various types of adrenal growth in vivo is poorly known. Therefore, the aim of this study was to investigate the adrenal transcriptome profile during the very specific compensatory adrenal growth, that is, the growth evoked by unilateral adrenalectomy.

## 2. Results

### 2.1. Unilateral Adrenalectomy-Induced Compensatory Adrenal Growth Increases the Weight of the Remaining Gland 24 and 72 h after Hemiadrenalectomy

**Figure 1:**
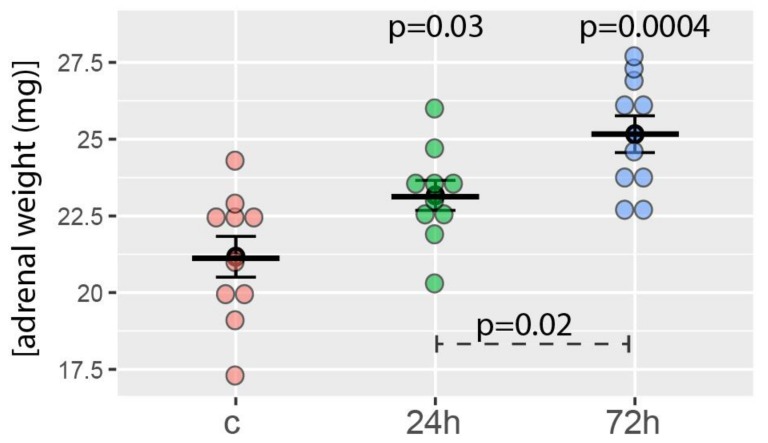
Right adrenal weight obtained from the control group (c, sham adrenalectomy, after 24 h) as well as from two experimental groups: right adrenals from 24 and 72 h after left unilateral adrenalectomy (*n* = 10/experimental group). Mean, standard error of the mean, and *p*-values of the Student’s *t*-test are shown. The *p*-values located above the experimental groups refer to a comparison with control. The *p*-value over the dashed line refers to the comparison between experimental groups (24 vs. 72 h after left unilateral adrenalectomy).

The average weight of the right adrenal glands of the control group was 21.1 ± 2.5 mg. As expected, the weights of the right adrenal glands of rats tested 24 and 72 h after hemiadrenalectomy increased statistically significantly. It amounted to 23.1 ± 1.5 and 25.2 ± 1.9 mg, respectively (Figure 1). The difference in the adrenal mass of both groups of rats subjected to hemiadrenalectomy was also statistically significant. The relevant *p*-values are shown in Figure 1.

### 2.2. Unilateral Adrenalectomy Exerts a Modest Influence on the Entire Adrenal Gland Gene Expression Profile

**Figure 2:**
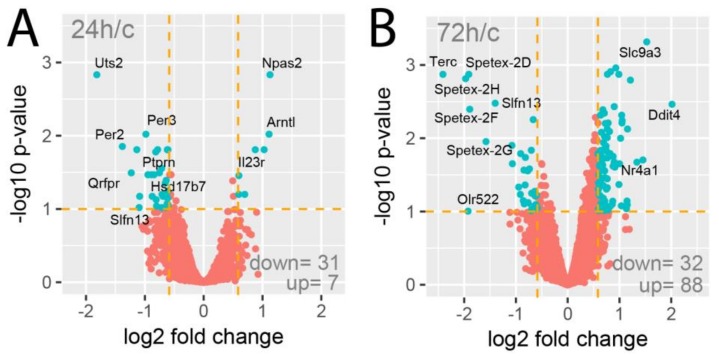
Volcano plots of total gene expression profiles of the right rat adrenals obtained 24 (**A**) or 72 h (**B**) after a left adrenalectomy in relation to adrenals from control rats. Each dot represents the mean expression (*n* = 5) of individual genes obtained from a microarray normalized dataset. The orange dotted lines (cut-off values) were established according to the following parameters: fold = |1.5| and *p*-value with FDR correction =10%. Genes above the cut-off lines have been considered as differentially expressed genes (DEG) and are shown as turquoise dots. The total numbers of DEG are presented in the bottom right corner of the graph. Ten of the most regulated genes are marked by their gene symbols.

The Affymetrix Rat Gene 2.1 ST Array Strips used in the current study allowed the simultaneous examination of the expression of 28,407 transcripts. As shown on the volcano plots, only a limited number of genes were affected by the experimental procedures applied (Figure 2). Despite the assumption of relatively low cut-off values (fold change |1.5| and *p*-value with FDR correction 10%), expression of only 38 genes (31 down, 7 up) was regulated in the 24-h vs. control group comparison (Figure 2A). Under identical assumptions, the total number of differentially expressed genes in the 72-h vs. control group comparison revealed 120 differentially expressed genes (32 down, 88 up; Figure 2B).

**Figure 3:**
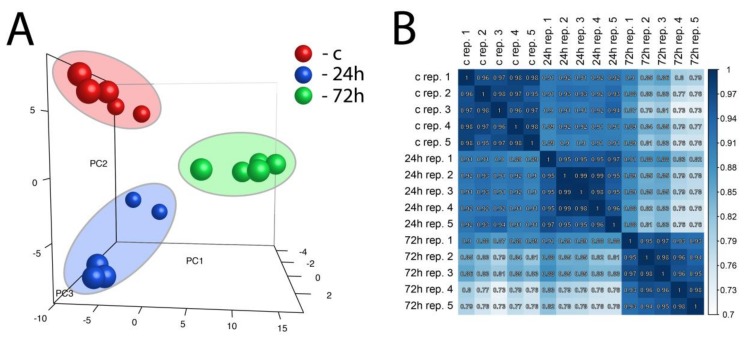
Principal component analysis (PCA) graph of a whole set of differentially expressed genes from all studied comparisons (24 h/c and 72 h/c) (**A**). Each dot represents one of the experimental sample assigned to the appropriate experimental group. (PC1: principal component 1; PC2: principal component 2; PC3: principal component 3). Pearson’s correlation coefficient analysis array (**B**). The correlation coefficient was calculated for all experimental groups.

Principal component analysis (PCA), as well as correlation coefficient analysis, was performed on a whole set of differentially expressed genes from all studied comparisons (Figure 3A,B). PCA enabled three independent clusters to be obtained that corresponded to our experimental group (control, 24 h, and 72 h). Taking into account the value of the correlation coefficient, as well as the position of the samples on the PCA graph, it is worth emphasizing that gene expression after 24 h of compensatory adrenal growth is more convergent with the control group than with compensatory adrenal growth after 72 h.

A full table with gene names, gene symbols, fold changes, and statistical estimations are included in the supplementary material (Table S1). The 20 genes from each of the experimental comparisons (24 h vs. control and 72 h vs. control) with the highest (10 genes) and lowest (10 genes) fold change values are presented in tabular format, displaying their gene symbol, gene name, fold change, and adjusted *p*-value (Table 1).

**Figure 4:**
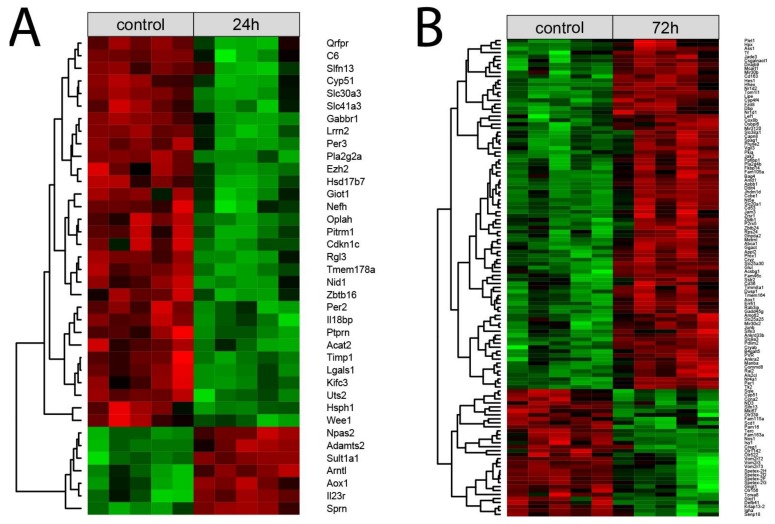
Heatmap with hierarchical clusterization of the differentially expressed genes in the right adrenals collected 24 h (**A**) or 72 h (**B**) after left adrenalectomy in relation to the right adrenals from sham-operated rats. Normalized signal intensity acquired from the microarray analysis is represented by color (green = higher expression; red = lower expression). Log2 signal intensity values for any single gene were resized to row Z-score scales.

Each normalized signal intensity from two sets of differentially expressed genes (24 h vs. control and 72 h vs. control) was grouped using a hierarchical clusterization algorithm. The results of such analysis are presented as a heatmap (Figure 4). Clusterization confirmed the presence of two superior clusters of the size corresponding to the number of up- and down-regulated genes. (Figure 4A,B, respectively).

### 2.3. Unilateral Adrenalectomy-Induced Compensatory Growth Affects the Expression of Genes Involved in Lipid and Steroid, Circadian Rhythm, Stress, and Cell Cycle Regulations

**Figure 5:**
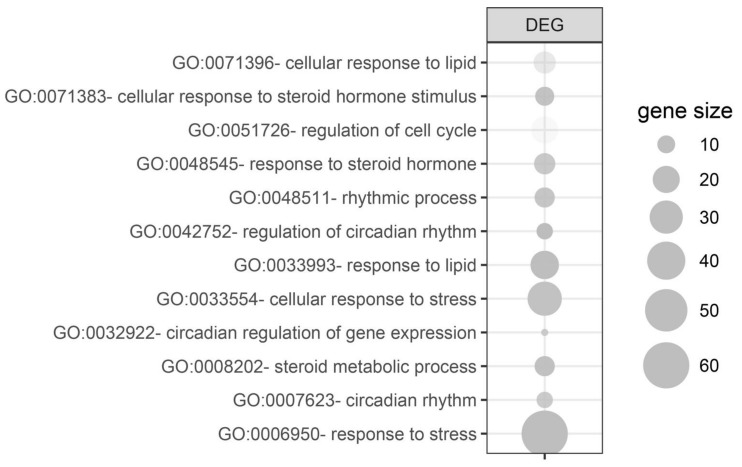
Gene ontological (GO) groups relevant to adrenal physiology, selected by searching the DAVID GOTERM BP database using all of the differentially expressed genes (both from 24 h/control and 72 h/control comparisons). The graphs show only the GO groups above the established cut-off criteria (*p* with correction <0.05, a minimal number of genes per group >5). The size of each bubble reflects the number of differentially expressed genes assigned to the GO terms. The transparency of the bubbles displays the *p*-values (more transparent is closer to the border of *p* = 0.05).

Due to the limited number of differentially expressed genes, assignment to the relevant gene ontological (GO) group was performed on all differentially expressed genes simultaneously (both from 24 h and 72 h groups vs. control). For this purpose, 158 differentially expressed gene names (38 from 24 h vs. control and 120 from 72 h vs. control) were used as a query for DAVID GOTERM BP database, searching with the assignment of differentially expressed genes to appropriate GO groups. Twelve processes relevant to adrenal gland physiology were isolated from them and are presented in Figure 5. These processes concerned the following GO groups: “GO:0071396—cellular response to lipid” (*n =* 14, *p-*value = 3.83 × 10^−2^); “GO:0071383—cellular response to steroid hormone stimulus“ (*n =* 11, *p-*value = 8.10 × 10^−3^); “GO:0051726—regulation of cell cycle” (*n =* 20, *p-*value = 4.65 × 10^−2^); “GO:0071383—cellular response to steroid hormone stimulus” (*n =* 11, *p-*value = 8.10 × 10^−3^); “GO:0048545—response to steroid hormone” (*n =* 13, *p-*value = 1.57 × 10^−2^); “GO:0048511—rhythmic process” (*n =* 12, *p-*value = 1.24 × 10^−2^); “GO:0042752—regulation of circadian rhythm” (*n =* 9, *p-*value = 2.08 × 10^−3^); “GO:0033993—response to lipid” (*n =* 22, *p-*value = 6.14 × 10^−3^); “GO:0071396—cellular response to lipid” (*n =* 14, *p-*value = 3.83 × 10^−2^); “GO:0033554—cellular response to stress” (*n =* 32, *p-*value = 8.89 × 10^−3^); “GO:0032922—circadian regulation of gene expression” (*n =* 6, *p-*value = 1.78 × 10^−2^); “GO:0008202—steroid metabolic process” (*n =* 12, *p-*value = 8.68 × 10^−3^); “GO:0042752—regulation of circadian rhythm” (*n =* 9, *p-*value = 2.08 × 10^−3^); “GO:0007623—circadian rhythm” (*n =* 9, *p-*value = 1.78 × 10^−2^); “GO:0006950—response to stress” (*n =* 60, *p-*value = 1.05 × 10^−4^); and “GO:0033554—cellular response to stress (*n =* 32, *p-*value = 8.89 × 10^−3^).

**Figure 6:**
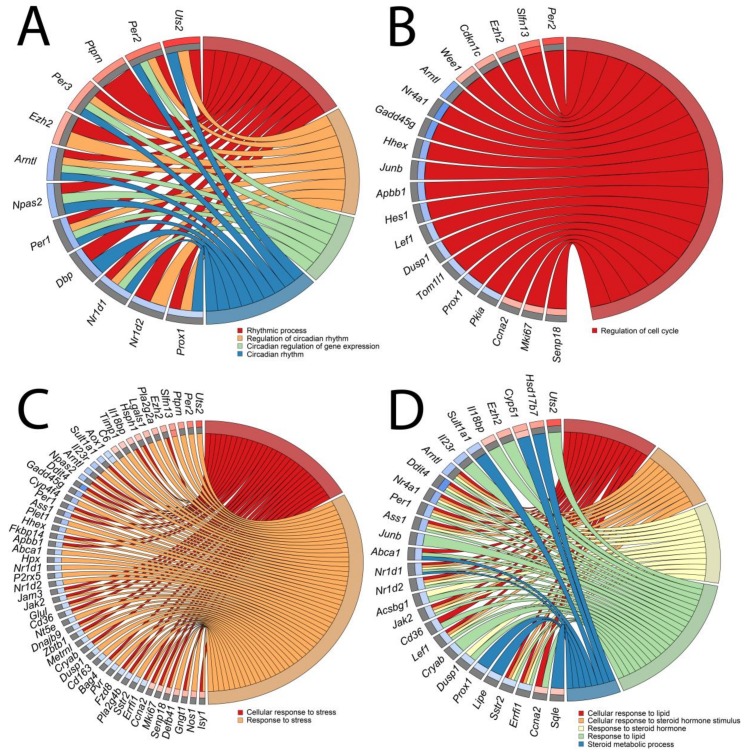
Circos plots of closely related GO terms ((**A**)—circadian rhythm, (**B**)—cell cycle, (**C**)—stress, (**D**)—lipid and steroid) and differentially expressed genes belonging to relevant GO terms. Symbols of DEG are presented on the left side of the graph with their fold change values mapped by color scale (red = higher expression; blue = lower expression, gray = genes below the cut-off criteria with no statistical difference). Values of fold change are shown separately for 24 h vs. control (external rectangles) and for 72 h vs. control comparisons (internal rectangles). Gene involvement in the GO terms was determined by colored connecting lines.

Due to the high similarities of certain GO groups, they were classified into four categories: (A) “circadian rhythm”; (B) “cell cycle”; (C) “stress”; and (D) “lipid and steroid” and presented as circos (circular genome data visualization) plots (Figure 6A–D, respectively). Symbols of genes constituting given GO term categories are shown. Relevant fold change values are presented by the color scale (red—up-regulated, blue—down-regulated in relation to the control group), where external rectangles relate to 24 h vs. control whilst internal rectangles refer to 72 h vs. control expression changes. Interestingly, apart from a few differentially expressed genes whose expressions were regulated both in the same way after 24 and 72 h (*Cyp 51*, *Slfn13*, *Aox1*), different genes forming specific GO categories were derived from 24 h vs. control and 72 h vs. control groups. In addition, the majority of genes from 24 h vs. control underwent positive stimulation whilst the expression of genes from 72 h vs. control was mostly down-regulated.

The expression of genes belonging to the “circadian rhythm” category show high dynamics of change. In comparison of 24 h/c five genes were up-regulated and two down-regulated whilst in the 72 h vs. control groups five genes were down-regulated (Figure 6A). Interestingly, unilateral adrenalectomy affected the expression of key genes of the circadian rhythm pathway, such as *Per1* (period circadian clock 1; down-regulated in 72 h vs. control), *Per2* (period circadian clock 2; up-regulated in 24 vs. control), *Per3* (period circadian clock 3; up-regulated in 24 vs. control), *Arntl* (aryl hydrocarbon receptor nuclear translocator-like protein 1; down-regulated in 24 vs. control), and *Nr1d1* (nuclear receptor subfamily 1 group D member; down-regulated in 72 vs. control).

The “cell cycle” category consisted of only one gene ontological group—“regulation of cell cycle” (Figure 6B). As before, the majority of differentially expressed genes in the 24 h vs. control comparison were up-regulated (five genes) and only one gene was down-regulated. In comparison with 72 h vs. control, the expression of four genes was up-regulated while eleven genes were down-regulated. Interestingly, we noticed that the classical marker of proliferation *Ki-67* (*mki67*) was not significantly changed in the 24-h to control comparison but it was up-regulated after 72 h of compensatory adrenal growth. *Ccna2* (Cyclin A2) gene was regulated in a similar manner while *Cdkn1c* (cyclin-dependent kinase inhibitor 1C) was up-regulated after 24 h of adrenal compensatory growth and remained unchanged at 72 h of the experiment.

The gene expression pattern of the “stress” category was notably changed during the experiment (Figure 6C). In this category, eleven genes were up-regulated and five were down-regulated in 24 h vs. control comparisons. Again, the opposite pattern was observed in the 72 h vs. control groups with seven up-regulated and 32 down-regulated genes.

In the “lipid and steroid” GO category (Figure 6D), the DEGs from 24 h vs. control groups were characterized by the following distribution: five genes were up-regulated and three genes were down-regulated. The opposite distribution of differentially expressed genes was observed in the 72 h vs. control groups, where three genes were up-regulated and eighteen were down-regulated. The results of our study did not reveal any differences in the expression of genes involved in the classical adrenal steroidogenesis pathway (*StAR*, *Cyp11a1*, *Cyp11b1, Cyp11b2*); however, we observed changes in the expression of genes involved indirectly in steroidogenesis, such as hormone-sensitive lipase (*Lipe*), whose expression was lowered at 72 h of compensatory growth.

Due to the ambiguous nature of the gene ontology structure, it is worth noticing that there were genes that belong to several described GO categories. Among others, this concerns *Uts2* (urotensin 2) gene, which is listed in the “circadian rhythm,” “stress,” and “lipid and steroid” GO categories.

## 3. Discussion

As mentioned in the introduction, the genetic basis of adrenal growth regulation in vivo is not well known and only a few publications have been devoted to this issue. Normal adrenal growth with age ensures that the adrenal mass and its secretory function is maintained during the changing body mass and changing demand for corticosteroids. As Dallman [1] points out, this type of growth is rather predictable and depends on both hyperplasia and hypertrophy of adrenocortical cells [2,3].

The unilateral adrenalectomy-induced compensatory adrenal gland growth has been known for almost 100 years [4]. This type of adrenal growth in vivo seems to be highly specific and depends mainly on afferent and efferent nerve impulses [1,5,6,7,8,9]. As demonstrated by Dallman et al. [1], neural reflex, responsible for compensatory adrenal growth, originates in one adrenal gland. This reflex, via afferent nerves, interneurons in or passing through the hypothalamus, and via efferent nerves from the hypothalamus, affects the contralateral gland. Probably, this nervous reflex signals the mass of the cortex of one adrenal gland to the other adrenal gland [1,10]. However, since the regeneration of adrenals occurs also in ectopic sites (for example adrenal transplants), it has been suggested that this reflex is not essential for regulating other types of adrenal growth in vivo. Unilateral adrenalectomy-induced compensatory adrenal growth primarily depends on the proliferation of adrenocortical cells [1,5]. In the rat, the removal of one adrenal is followed by two waves of adrenocortical cell proliferation in the remaining gland at days 1 and 3 after surgery [11]. This type of adrenal growth is not blocked by hypophysectomy or glucocorticoid administration (for review see [1]). In this respect, it should be emphasized that biologically active neuropeptides (for example, neuromedin U) can modulate this type of adrenal growth [12].

In the available literature, we have not found a publication on the global expression of adrenal genes in the case of compensatory growth induced by hemiadrenalectomy. The present study revealed that in the rat, compensatory adrenal growth induced by unilateral adrenalectomy exerts a limited influence on adrenal gland gene expression profiles, even with the assumption of relatively low cut-off values (fold change: |1.5|; *p*-value with FDR correction: 10%). Using the Affymetrix microarray system, in the remaining adrenal glands at 24 and 72 h of the experiment, we showed only 155 significantly regulated genes. After bioinformatics analyses, these genes could be grouped into four significantly enriched GO categories. These categories are “circadian rhythm,” “cell cycle,” “stress,” and “lipid and steroid”. It is worth noticing that neural adrenal stimulation affects all of them. Splanchnic nerve stimulation enhances the production of glucocorticoids in response to ACTH, as well as stimulates basic adrenocortical steroidogenesis [13,14]. Sympathetic innervations also play an essential role in adrenal diurnal steroidogenesis variations and participate in the regulation of adrenocortical cell division [6,15].

The present study demonstrated that in unilateral adrenalectomy-induced adrenal growth, the gene expression profile of the rat in the remaining adrenal gland differs significantly in the groups studied 24 and 72 h after unilateral adrenalectomy Most of the adrenal differentially expressed genes were up-regulated at 24 h of the experiment, while they were down-regulated 72 h after surgery. This indicates high dynamic changes in adrenal gene expression in response to the removal of the contralateral adrenal gland.

In this study, the most significant changes in gene expression during compensatory adrenal growth were observed in the GO category “circadian rhythm.” We found that unilateral adrenalectomy significantly affected core genes regulating the circadian rhythm, which are responsible for the daily rhythmical secretion of adrenal hormones. Currently, it is well known that adrenal circadian rhythms are controlled by three factors: (I) hypothalamic–pituitary–adrenal (HPA) axis, (II) hypothalamic suprachiasmatic nucleus (SCN) acting via the autonomic nervous system, and (III) local intra-adrenal circadian clocks [16]. It is generally accepted that in mammals, SCN acts as the master circadian pacemaker [17,18]. Interestingly, the influence of SCN neurons on adrenal circadian rhythm goes through the splanchnic nerve that is also activated during compensatory adrenocortical growth [17,19]. Furthermore, hypophysectomy does not affect either the regulation of circadian rhythm or the compensatory adrenocortical growth [20,21]. On the molecular level, the circadian mechanism composed of transcriptional activators consists of a Clock and Arntl (Bmal1) heterodimer that binds E-box enhancer elements upstream of cryptochrome (Cry1 and Cry2), period (Per1, Per2, Per3), and Nr1d1 (Rev-erb-alfa) and other clock-controlled output genes [22,23]. Subsequently, Cry and Per proteins are imported into the nucleus to inhibit the activity of Clock⁄Arntl, and thus repress their own transcription by interacting in a feedback loop with Clock/Arntl complexes. The *Nr1d1* that encodes Rev-erb-alfa protein also acts as a repressor of Arntl1 transcription and, therefore, forms an additional negative loop to the circadian cycle [20]. Our current studies reveal that after 24 h compensatory adrenocortical growth, expression of *Arntl* was down-regulated whilst *Per2* and *Per3* were up-regulated. After 72 h expression of *Per 1*, *Nr1d1* and its paralog *Nr1d2* were down-regulated in relation to control adrenals. Our results are in line with data of Oster et al. [24] who revealed that the expression of the majority of canonical clock genes—*Arntl* (*Bmal1*), *Cry1, Per1, Per2, Per3, and Nr1d1* (Rev-erb-alfa)—undergo rhythmical changes in the mice adrenal gland. *Clock* and *Cry2* transcripts were also detected but did not show obvious circadian rhythm changes. The expression of these genes also did not change in our studies. Our studies do not confirm the expression changes in *Cry1*; however, expression of this gene was slightly elevated at 72 h (fold = 1.4), but did not reach statistical significance (adj. *p* = 0.37). Considering the above-described results, we assume that unilateral adrenalectomy-induced compensatory adrenal growth, through the splanchnic nerve activation, causes a shift in the expression of core genes of the circadian clock. Circadian pattern of expression may affect approximately 5% of the mice adrenal transcriptome, including genes involved in cholesterol uptake, regulators of steroidogenesis, proliferation, and cell cycle regulation [25]. In our studies, less than 1% of genes were differentially regulated. However, we cannot exclude circadian rhythm-dependent regulation of other genes during the studied time of compensatory adrenal growth. It should be noted that although the circadian clock is often described as a transcription-affected mechanism, many posttranslational modifications (PTMs) are important for its physiological function, including phosphorylation, ubiquitination, sumoylation, and acetylation [26]. The influences of unilateral adrenalectomy on circadian rhythm-dependent posttranslational modifications in the remaining gland are waiting for further research.

Our observations are also in line with the data reported and reviewed by Dallman [1]. She showed that the rate and degree of adrenal response to unilateral adrenalectomy are very sensitive to light cycles. It had been suggested that in unilateral adrenalectomy-induced compensatory growth, constant light and pineal factors act to inhibit the neural stimulation of the remaining adrenal gland. In the current literature, there are no direct reports about the inhibitory effect of melatonin on unilateral adrenalectomy-induced compensatory adrenal growth. However, the demonstration of the expression of key genes controlling adrenal gland rhythm [24] and the significant changes in their expression observed in the current research seem to support the suggestions described earlier [1]. It should be emphasized that in the examined adrenal glands after both 24 h and 72 h, we observed unaltered expression of melatonin receptors (*Mtnr1a*: fold 24 h/c = −1.10, fold 72 h/c = −1.05, adj. *p* 24 h/c = 0.92, adj. *p* 72 h/c = 0.90; *Mtnr1b*: fold 24 h/c = −1.14, fold 72 h/c = −1.09, adj. *p* 24 h/c = 0.91, adj. *p* 72 h/c = 0.86). Rhythmic expression of melatonin and several clock genes in the mouse, rat, and primate adrenal glands, as well as in adrenal explants, has previously been reported [20,27,28,29]. It should be emphasized, however, that the rhythmic expression of clock genes were observed in melatonin-deficient C57BL/6J mice [30]. These observations suggest that the rhythmic expression of “clock genes” in the adrenal gland may be independent of melatonin.

Unilateral adrenalectomy-induced compensatory adrenal growth is characterized by a prompt increase in weight and DNA and RNA in the remaining adrenal; however, the RNA/DNA ratio does not change [31,32,33]. Such changes suggest that compensatory adrenal growth in rats is of a proliferative type. It is assumed that two waves of parenchymal cell proliferation occur during compensatory adrenocortical growth at 24 and 72 h after surgery [11,12]. Thus, in the performed study at both time points after unilateral adrenalectomy, we should expect a similar pattern of expression of genes belonging to the “regulation of cell cycle” GO term. However, the level of gene expression of this ontological group varies significantly at both examined time points. This pattern of expression of cell cycle control genes may indicate high dynamics of changes in proliferation (cell cycle) control during compensatory adrenal growth. The obtained data also suggests that during this period, molecular mechanisms controlling this type of adrenal growth are changing. Unilateral adrenalectomy-induced compensatory adrenal growth in the rat is primarily regulated by nervous reflexes but probably other factors, including humoral factors, also play a role. It is worth noting that the expression level of the classic proliferation marker gene *Ki-67* (*mki67*) was not changed at 24 h of compensation but increased at 72 h of the experiment.

Several studies point to the supportive role of ACTH or other POMC-derived peptides in the early stage of the compensatory adrenal growth. It has been shown that pro-gamma MSH stimulates proliferation of the remaining adrenal at 24 h of compensatory growth [33]. Moreover, adrenal growth-stimulating factors other than POMC-derived peptides may also play a supportive role at the early stages of adrenal compensatory growth in an auto- and paracrine manner. In this context, the roles of basic fibroblast growth factor and neuromedin U have been described [12,34]. Interestingly, in our studies, we show that the expression of the urotensin 2 gene (*Uts2*)—coding one of the potent vasoconstrictive peptides—was significantly elevated at 24 h of compensatory adrenocortical growth. Intra-adrenal blood flow rate is highly controlled by a number of different neural and hormonal stimuli in which activation of the splanchnic nerve, as well as ACTH, plays a crucial role [6]. The results of our unpublished studies indicate that *Uts2* expression is strongly up-regulated by ACTH. Therefore, we can assume that increased *Uts2* expression during the early stages of adrenal compensatory growth can be exerted by ACTH.

The gene expression pattern of the “stress” category was notably changed in the course of compensatory adrenal growth. As expected, the expression of most of the stress category genes was up-regulated at 24 h of the experiment while down-regulated at 72 h.

In our research, rather surprising results are evident concerning genes belonging to the “lipid and steroid” GO group. Unilateral adrenalectomy changed the expression of only a small number of these genes. It is interesting that the expression of genes directly involved in the classical pathway of steroidogenesis (*StAR, Cyp11a1, Cyp11b1, Cyp11b2*) did not change in the enlarged adrenal gland. Unlike these genes, genes indirectly involved in steroidogenesis, such as hormone-sensitive lipase (*Lipe*), were reduced at 72 h in the experiments. *Lipe* is a major cholesterol hydrolase of the adrenal glands. It is also defined as a key enzyme involved in steroid hormone synthesis. Down-regulation of *Lipe* gene indicates that the efficiency of adrenal steroidogenesis may be reduced at 72 h of adrenal compensatory growth. [35,36,37].

## 4. Materials and Methods

### 4.1. Animals and Experiments

Adult male Wistar rats (12 weeks old, final body weight 160–180 g) were obtained from the Laboratory Animals Breeding Center, Department of Toxicology, Poznan University of Medical Sciences, Poznan, Poland. Animals were kept under the standard conditions of 14:10 h light–dark cycle (illumination onset at 6:00) at 23 °C constant temperature. The animals had free access to standard diet and water. All procedures described below were approved by the Local Ethics Committee for Animal Research (Poznan, Poland), permission number: LKE-11/2015. Maximum efforts were made to minimize the number of animals and their suffering.

### 4.2. Reagents

If not otherwise stated, all reagents were obtained from Sigma-Aldrich (Merck KGaA, Darmstadt, Germany) or Avantor Performance Materials Poland S.A. (Gliwice, Poland).

### 4.3. Unilateral Adrenalectomy

Surgical removal of the left adrenal gland (left hemiadrenalectomy) or sham operation was performed under standard ketamine (100 mg/kg, i.p.; (Ketanest^®^, Pfizer Europe, Sandwich, UK) and xylazine (10 mg/kg, i.p.; Sedazin^®^, Biowet, Puławy, Poland) anesthesia. Hemiadrenalectomy was accomplished via the classic dorsal approach [4]. In the control group (sham hemiadrenalectomy) adrenals were visualized but not manipulated. Sham-operated animals were decapitated 24 h post-surgery while hemiadrenalectomized rats were decapitated 24 and 72 h post-surgery. In each group, there were 10 rats. The surgical procedures, as well as the completion of the experiment, were carried out between 10 and 11 a.m. The experiment was planned in such a way that all animals were sacrificed on the same day. After decapitation, the adrenals were collected, freed from the adjacent adipose tissue, weighed, and stored in RNAlader at −70 °C for further analysis.

### 4.4. RNA Extraction

Two adrenals from the same experimental group were pooled together to give five samples per group. Isolation of the RNA was performed using the modified Chomczynski method with the use of TRI Reagent (Sigma, St. Louis, MO, USA) and subsequent purification by RNeasy Mini Elute Cleanup Kit (Qiagen, Hilden, Germany), according to the manufacturer’s guidelines. The concentration of the total RNA was assessed spectrophotometrically (260 nm). The purity of the isolated RNA was determined by applying the 260/280 nm absorption ratio, which was equal to or greater than 1.8 (NanoDrop ND-1000 spectrophotometer; Thermo Fisher Scientific, Inc., MA, USA). The quality and integrity of the RNA were also verified in a Bioanalyzer 2100 (Agilent Technologies, Inc., Santa Clara, CA, USA). The resulting RNA integrity numbers (RINs) ranged from 8.5 to 10 with a mean of 9.2 (Agilent Technologies, Inc., Santa Clara, CA, USA). Total RNA in a concentration of 50 ng/µL was used for the microarray experiment.

### 4.5. Microarray Expression Study

The microarray study was carried out according to the previously described procedure [38,39,40,41]. The complete procedure for preparing RNA for hybridization was performed using the GeneChip WT PLUS Reagent Kit (Affymetrix, Santa Clara, CA, USA). Two-step cDNA synthesis reaction was carried out using 100 ng of total RNA and random primers extended by the T7 RNA polymerase promoter sequence. The synthesis of cRNA was performed by in vitro transcription under the following conditions: 16 h, 40 °C. Then, cRNA was purified and re-transcribed into cDNA, which was biotin labeled and fragmented using the Affymetrix GeneChip WT Terminal Labeling and Hybridization kit (Affymetrix, Santa Clara, CA, USA). These biotin-labeled fragments of cDNA were hybridized by the Affymetrix Rat Gene 2.1 ST Array Strip (20 h, 48 °C). After that, the microarrays were subjected to a washing and staining procedure, according to the technical protocol, using the Affymetrix GeneAtlas Fluidics Station (Affymetrix, Santa Clara, CA, USA). The array strips were scanned using an Imaging Station of GeneAtlas System (Thermo Fisher Scientific, MA, USA). The preliminary analysis of the scanned chips was carried out by means of Affymetrix GeneAtlas Operating Software (Affymetrix, Santa Clara, CA, USA). The quality of the gene expression data was verified using the quality control criteria established by the software.

### 4.6. Microarray Data Analysis

The generated CEL files were subjected to further analysis using the R statistical language and Bioconductor package with the relevant Bioconductor libraries. The Robust Multiarray Average (RMA) normalization algorithm implemented in the “Affy” library was used for normalization, background correction, and calculation of the expression values of all of the examined genes [42]. Assigned biological annotations were taken from “pd.ragene.2.1.st” library that was used for the mapping of normalized gene expression values with their symbols, gene names, and Entrez IDs, leading to a complete gene data table. Differential expression and statistical assessment were determined by applying the linear models for microarray data implemented in the “limma” library [43]. Normalized gene expression datasets were visualized on volcano plots with relation to cut-off criteria. The accepted cut-off criteria were based on both differences in expression fold change (FC) greater than abs. 1.5 and 10% false discovery rate (FDR) correction. Genes that fulfilled the selection criteria were considered significantly different and are marked in turquoise on the volcano plots. The whole sets of differentially expressed genes from all studied comparisons were subjected to principal component analysis (PCA), as well as correlation coefficient analysis. For this purpose, “pca3d” and “corrplot” Bioconductor libraries were used [44,45]. Differentially expressed genes were also subjected to a hierarchical clusterization algorithm and visualized as heat maps. Other detailed analyses were conducted only on differentially expressed gene sets.

### 4.7. Assignment of Differentially Expressed Genes to Relevant Gene Ontology (GO) Terms

All differentially expressed genes from both comparisons (24 h to control and 72 h to control) were subjected to functional annotation and clusterization using the DAVID (Database for Annotation, Visualization, and Integrated Discovery) bioinformatics tools [46]. Gene symbols of differentially expressed genes were uploaded to DAVID by the “RDAVIDWebService” Bioconductor library [47], where DEGs were assigned to relevant GO terms, with subsequent selection of significantly enriched GO terms. The *p*-values of selected GO terms were corrected using Benjamini–Hochberg false discovery rate method described as adjusted *p*-values [48]. GO groups essential for adrenal physiology with adjusted *p*-values below 0.05 were visualized using bubble plots. Details of the genes belonging to particular GO terms with their fold change values were presented as circus plots using “GOplot” library [49].

### 4.8. Statistics

Differences in adrenal mass between individual experimental groups were evaluated by a Student’s *t*-test. In the case of data obtained from microarrays, differences were evaluated by statistical programs included in particular bioinformatics analyses.

## 5. Conclusions

The presented study concerns the determination of the adrenal transcriptome profile at two critical stages of adrenal compensatory growth. We found that compensatory adrenal growth induced by unilateral adrenalectomy had a limited influence on the adrenal gland gene expression profile. In our comprehensive approach, we used the whole adrenal gland to define transcriptome profiles and, thereby, pronounced changes in expression may have been underestimated by the predominance of unresponsive cells. Further detailed transcriptome studies are required based on separated adrenocortical zones and medulla. Nevertheless, we identified 155 genes that were significantly regulated both after 24 and 72 h. In the rat, unilateral adrenalectomy-induced compensatory adrenal growth leads to the most significant changes in the expression of key genes regulating the circadian rhythm. Our results confirm also that regulation of compensatory adrenal growth is under complex and multifactorial control, with a pivotal role of neural regulatory mechanisms and a supportive role of other components.

## Figures and Tables

**Table 1:** List of 20 genes with the highest (10 genes) and lowest (10 genes) fold change obtained from the datasets of differentially expressed genes from 24 h/c (left) and 72 h/c (right) comparisons.

24 h/c				72 h/c			
Gene Symbol	Gene Name	Fold Change	Adj. *p* Value	Gene Symbol	Gene Name	Fold Change	Adj. *p* Value
*Uts2*	urotensin 2	3.52	0.00147	*Terc*	telomerase RNA component	5.31	0.00134
*Per2*	period circadian clock 2	2.61	0.01402	*Spetex-2H*	Spetex-2H protein	3.92	0.00154
*Qrfpr*	pyroglutamylated RFamide peptide receptor	2.35	0.03213	*Olr522*	olfactory receptor 522	3.79	0.09894
*Ptprn*	protein tyrosine phosphatase, receptor type, N	2.20	0.01550	*Spetex-2D*	Spetex-2D protein	3.76	0.00134
*Slfn13*	schlafen family member 13	2.14	0.09564	*Spetex-2F*	Spetex-2F protein	3.71	0.00402
*Hsd17b7*	hydroxysteroid (17-beta) dehydrogenase 7	2.13	0.06722	*Spetex-2G*	Spetex-2G protein	2.98	0.01111
*Per3*	period circadian clock 3	1.98	0.00954	*Slfn13*	schlafen family member 13	2.64	0.00333
*Cyp51*	cytochrome P450, family 51	1.93	0.03407	*Igha*	immunoglobulin heavy chain, alpha	2.11	0.01254
*Ezh2*	enhancer of zeste 2 polycomb repressive complex 2 subunit	1.85	0.03407	*Vom2r73*	vomeronasal 2 receptor, 73	2.10	0.02229
*Giotl*	gonadotropin inducible ovarian transcription factor 1	1.83	0.06722	*Vom2r72*	vomeronasal 2 receptor, 72	1.93	0.04645
*Cyp4f4*	cytochrome P450, family 4, subfamily f, polypeptide 4	−1.51	0.31696	*Hhex*	hematopoietically expressed homeobox	−2.07	0.00573
*Adamts2*	ADAM metallopeptidase with thrombospondin type 1 motif, 2	−1.51	0.03504	*Pletl*	placenta expressed transcript 1	−2.20	0.09155
*Cox8b*	cytochrome c oxidase, subunit Vlllb	−1.52	0.23584	*Ass1*	argininosuccinate synthase 1	−2.21	0.07197
*MiR3120*	microRNA miR-3120	−1.54	0.14294	*Osbpl6*	oxysterol binding protein-like 6	−2.22	0.08097
*Ass1*	argininosuccinate synthase 1	−1.58	0.64063	*Perl*	period circadian clock 1	−2.23	0.00748
*Aox1*	aldehyde oxidase 1	−1.62	0.06324	*Cyp4f4*	cytochrome P450, family 4, subfamily f, polypeptide 4	−2.32	0.00161
*Sult1a1*	sulfotransferase family, cytosolic, 1A, phenol-preferring, member 1	−1.84	0.01553	*Gadd45g*	growth arrest and DNA-damage-inducible, gamma	−2.53	0.02125
*H23r*	interleukin 23 receptor	−2.04	0.01550	*Nr4a1*	nuclear receptor subfamily 4, group A, member 1	−2.74	0.01979
*Arntl*	aryl hydrocarbon receptor nuclear translocator-like	−2.16	0.00954	*Slc9a3*	solute carrier family 9, subfamily A (NHE3, cation proton antiporter 3), member 3	−2.88	0.00048
*Npas2*	neuronal PAS domain protein 2	−2.18	0.00147	*Ddit4*	DNA-damage-inducible transcript 4	−4.04	0.00344

## Supplementary Materials

Supplementary materials can be found at http://www.mdpi.com/1422-0067/19/4/1111/s1. Table S1. Excel document file with normalized log2 signal intensities from control, sham operated group (control 1–5) and two experimental groups—right adrenals from rats autopsied 24- and 72-h after left unilateral adrenalectomy (24 h 1–5 and 72 h 1–5). Gene symbols, names, fold changes (fold 24 h/c, fold 72 h/c), *p*-values (*p* 24 h/c, *p* 72 h/c) and *p*-values corrected by FDR (adj. *p* 24 h/c, adj. *p* 72 h/c) are shown.

## Abbreviations

ACTH	adrenocorticotropic hormone
DEG	differentially expressed genes
FDR	false discovery rate
GO	gene ontology
DAVID	database for annotation, visualization and integrated discovery
BP	biological process
Cyp51	cytochrome P450, family 51, subfamily A, polypeptide 1
Slfn13	Schlafen family member 13
Aox1	aldehyde oxidase 1
Arntl	aryl hydrocarbon receptor nuclear translocator-like protein 1
Nr1d1	nuclear receptor subfamily 1 group D member 1
Nr1d2	nuclear receptor subfamily 1 group D member 2
Ki-67	marker of proliferation Ki-67
Ccna2	cyclin A2
Cdkn1c	cyclin-dependent kinase inhibitor 1C
StAR	steroidogenic acute regulatory protein
Cyp11a1	cytochrome P450 family 11 subfamily A member 1 (cholesterol 20–22 desmolase)
Cyp11b1	cytochrome P450 family 11 subfamily B member 1
Cyp11b2	cytochrome P450 family 11 subfamily B member 2 (aldosterone synthase)
Lipe	hormone-sensitive lipase
Uts2	urotensin 2
Y1	mouse adrenocortical cell line
Clock	Clock circadian regulator
Bmal1	brain and muscle ARNT-like 1
Cry1	cryptochrome circadian clock 1
Cry2	cryptochrome circadian clock 2
Per1	period circadian clock 1
Per2	period circadian clock 2
Per3	period circadian clock 3
C57BL/6J	inbred strain of laboratory mouse
POMC	pro-opiomelanocortin
MSH	melanocyte-stimulating hormone (melanotropin)
CEL	Affymetrix data file format
RMA	robust multi-array average
FC	fold change
